# Genetic diversity among five T4-like bacteriophages

**DOI:** 10.1186/1743-422X-3-30

**Published:** 2006-05-23

**Authors:** James M Nolan, Vasiliy Petrov, Claire Bertrand, Henry M Krisch, Jim D Karam

**Affiliations:** 1 Department of Biological Sciences, University of New Orleans, 2000 Lakeshore Dr., New Orleans, LA 70148, USA; 2 Department of Biochemistry, Tulane University Health Sciences Center, 1430 Tulane Ave., New Orleans, LA 70112, USA; 3 LMGM-CNRS UMR 5100,118, route de Narbonne, 31062 Toulouse cedex 09, France

## Abstract

**Background:**

Bacteriophages are an important repository of genetic diversity. As one of the major constituents of terrestrial biomass, they exert profound effects on the earth's ecology and microbial evolution by mediating horizontal gene transfer between bacteria and controlling their growth. Only limited genomic sequence data are currently available for phages but even this reveals an overwhelming diversity in their gene sequences and genomes. The contribution of the T4-like phages to this overall phage diversity is difficult to assess, since only a few examples of complete genome sequence exist for these phages. Our analysis of five T4-like genomes represents half of the known T4-like genomes in GenBank.

**Results:**

Here, we have examined in detail the genetic diversity of the genomes of five relatives of bacteriophage T4: the *Escherichia coli *phages RB43, RB49 and RB69, the *Aeromonas salmonicida *phage 44RR2.8t (or 44RR) and the *Aeromonas hydrophila *phage Aeh1. Our data define a core set of conserved genes common to these genomes as well as hundreds of additional open reading frames (ORFs) that are nonconserved. Although some of these ORFs resemble known genes from bacterial hosts or other phages, most show no significant similarity to any known sequence in the databases. The five genomes analyzed here all have similarities in gene regulation to T4. Sequence motifs resembling T4 early and late consensus promoters were observed in all five genomes. In contrast, only two of these genomes, RB69 and 44RR, showed similarities to T4 middle-mode promoter sequences and to the T4 *motA *gene product required for their recognition. In addition, we observed that each phage differed in the number and assortment of putative genes encoding host-like metabolic enzymes, tRNA species, and homing endonucleases.

**Conclusion:**

Our observations suggest that evolution of the T4-like phages has drawn on a highly diverged pool of genes in the microbial world. The T4-like phages harbour a wealth of genetic material that has not been identified previously. The mechanisms by which these genes may have arisen may differ from those previously proposed for the evolution of other bacteriophage genomes.

## Background

The T4-like phages are a diverse group of lytic bacterial myoviruses that share genetic homologies and morphological similarities with the well-studied coliphage T4 [1,2]. These phages provide an attractive model for the study of comparative genomics and phage evolution for several reasons: They possess relatively large dsDNA genomes that vary widely in size (~160–250 kb) and genetic composition. They contain host-like functions, such as nucleotide metabolism and a DNA replisome (reviewed in [3]). They experience different evolutionary constraints due to their lytic life cycle than do either their bacterial host or lysogenic bacteriophages. They exist under less stringent genomic size constraints than, for example, the lambdoid phages [4]. T4 has a terminally redundant genome [5] that replicates by a recombination-primed replication pathway. The efficient and promiscuous T4-encoded recombination machinery [6] may generate a high degree of evolutionary diversity, via both homologous and non-homologous recombination between this phage genome and that of bacterial hosts or other phages. Thus the characteristics of the T4-like genome, its mechanism of replication, and the interactions with cellular hosts suggest that the T4-like phages constitute a natural crucible for the acquisition, evolution and dispersal of genetic information in the microbial world.

We present here a bioinformatics analysis of the genome sequences of five T4-like bacteriophages. These phages include three coliphages (RB69, RB49 and RB43), and two *Aeromonas *phages (44RR2.8t and Aeh1). Our results complement and extend those previously reported from the coliphage T4 [3], the *Vibrio *phage, KVP40 [7], and from the marine cyanophages S-PM2 [8], P-SSM2 and P-SSM4 [9]. Our data identify a conserved core of T4-like genes found in all of these genomes, including some conserved ORFs of unknown function. One of the most striking findings is the presence of large numbers of novel open reading frames (ORFs), most of which have no significant match in GenBank. Both conserved and nonconserved regions of the genomes include sequence motifs resembling T4 promoters. Thus, it appears that both core and novel genes are co-ordinately expressed in a manner similar to that of T4. We compare the possible origins of the novel regions of the T4 genome with those proposed for other phages.

## Results

### Genome overview

We have analyzed five complete genome sequences of phylogenetically distant T4-like bacteriophages. This analysis is the first part of an ongoing comparative genomics project on T4-like phages. At present this project has generated single contiguous sequences for 12 divergent T4-like genomes. Of these sequences, five genomes were selected for in depth analysis on the basis of their phylogenetically diversity [10]. Among completed genomes that are not dealt with here are the *Aeromonas *phages 31 and 25, since they are both close relatives of 44RR2.8t and thus do not add significantly to the sequence diversity of the group. Five other genomes are considered draft quality (coliphages RB16 and phi-1, *Vibrio *phage nt-1, *Acinetobacter *phage 133, and *Aeromonas *phage 65) and are not included in this analysis but are available through the Tulane T4-like Genome Website . The five genomes presented here share between 61 and 67 percent amino acid similarity to each other among ~100 conserved open reading frames. T4 is most closely related to RB69, with which it shares 81% amino acid similarity over 207 ORFs. T4 exhibits about the same level of similarity to the other 4 genomes as they do to each other.

A summary of this analysis is presented in Table 1. The sizes of these five genomes range between 164 kb and 233 kb. The genome of Aeh1 had been predicted to be significantly larger than the other genomes, based on pulse field gel electrophoresis of genomic DNA [10]. This genome (233234 bp) is in fact nearly 40% larger than the average of T4 and the other four genomes presented here; the genomes of KVP40 [7] and P-SSM2 [9] are larger still (244 kb and 252 kb, respectively). All genomes have low %GC, although to a lesser degree than T4. ORFs were identified using GeneMarkS [11,12] and ORFs orthologous to T4 genes were identified by blastp mutual best hits to predicted proteins in the GenBank accession for the T4 genome. The probable significance of matches was assessed by expected value (E-value) scores. Most ORFs scored well below the 10^-4 ^cutoff for significant matches. A conserved core of 82 ORFs (T4-like genes) was found in all 5 genomes analysed here. There are 106 T4-like genes conserved among at least 4 of these 5 genomes; Aeh1 shared the fewest of these conserved genes (94) and the average similarity of the T4 orthologs of the conserved genes was lowest in this phage as well (49%). The conserved genes are generally clustered in several large blocks throughout each genome. Interspersed between these conserved blocks are segments containing blocks of predicted novel ORFs, most of which are unique to the genome that harbours them. Novel ORFs represent between 20% and 54% of the total coding capacity of the 5 genomes analyzed.

**Table 1:** Summary of T4-like genome sequences determined in comparison with T4

**Genome**	**Size (%GC)**	**# ORFS (% of genome)**	**# tRNAs**	**# T4-like ORFs (% of all)**	**#novel ORFs**
T4	168,904 (35.0%)	273 (95.9%)	8	209 (76.6%)	64
RB69	167,560 (37.6%)	273 (94.0%)	2	208 (77.7%)	65
RB49	164,018 (40.5%)	272 (94.5%)	0	121 (44.5%)	151
Aeh1	233,234 (42.8%)	332 (91.6%)	24	104 (31.3%)	228
RB43	180,500 (43.2%)	292 (94.2%)	1	114 (39.0%)	178
44RR 2.8t	173591 (44.0%)	253 (92.8%)	16	116 (45.8%)	137

The number of ORFs for T4 is from the GenBank accession but does not include 7 alternative translation products included within some ORFs. The number of ORFs predicted for T4 by GeneMarkS was 266 (93.1% of the genome length). tRNAs were predicted by tRNAscan-SE. The number of T4-like ORFs is the number of ORFs conserved in T4 and at least one of the other genomes studied. The remainder of ORFs in each genome are novel ORFs.

### Conserved genes and ORFs

**Figure 1:**
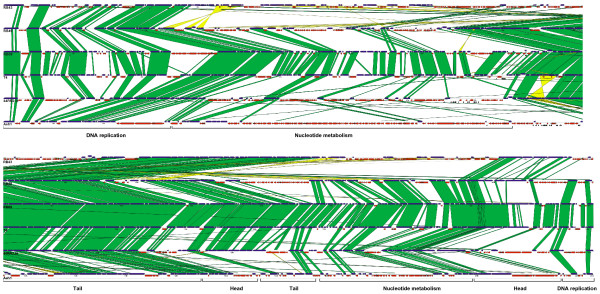
**Blast alignment of T4-like genomes**. Conserved T4-like genes are displayed as blue arrows, novel ORFs are shown as red arrows, tRNAs as black arrowheads. Pairwise tblastx similarities between genomes are indicated by green boxes. Similarities separated by less than 90 bp were combined for visual clarity. Yellow regions indicate similarities found in inverted orientation between genomes.

The conserved genes are generally localized in large clusters. The gene order among the clusters is highly collinear between most phages, as depicted in Figure 1: a higher resolution version is also available (see additional file 1). In T4, early and middle expressed genes are transcribed in a leftward direction (counterclockwise on the circular map), while late genes are primarily transcribed in the opposite direction. The genomes of RB69, RB49, and 44RR display a high degree of synteny with T4 and maintain essentially all of the clustering of related genes seen in T4. Synteny with T4 conserves the gene orientation with respect to time of expression during the infectious cycle. The genome of Aeh1 is also syntenous with T4, although small rearrangements of individual genes can be seen in Figure 1. Only RB43, with at least two substantial genome rearrangements, displays a significant break in synteny with T4 and the other T4-like phage genomes. The predicted transcription pattern appears more complex for RB43, with smaller clusters of genes predicted to be co-transcribed and some orthologs of T4 early and middle genes are transcribed from the opposite strand used in T4 [13]. A discussion of genes conserved in all T4-like phages can be found in a companion manuscript [13], as well as an earlier work [9].

The T4 genome has 132 predicted ORFs of unknown function. Eleven of these ORFs are conserved among the five T4-like genomes and orthologs to 93 T4 ORFs are found in at least one of these genomes. Although the conserved ORFs were not identified as essential in T4 by genetic methods [14], their preservation among phages suggests that they must be advantageous for survival in nature. In most instances the functions provided by these conserved ORFs remains obscure, but matches to Pfam motifs provide some clues about the function for a few of these ORFs, as shown in Table 2. For example, ORF *vs.6 *has a highly significant match to the Gly_radical Pfam accession, which is also found in the *nrdD *anaerobic nucleotide reductase. Thus, the *vs.6 *gene product may play a role in phage-induced nucleotide metabolism. Another conserved ORF, *vs.1*, exhibits marginally significant similarity to the SLT lytic transglycosylase domain, suggesting some role in cell lysis. These results corroborate PSI-BLAST matches previously reported for the T4 *vs.1 *and *vs.6 *ORFs to lysozyme and glycyl radical domains [15]. Overall, the match of *vs.1 *to the SLT domain is conserved; four of the six phage *vs.1 *orthologs match SLT with E value <0.05 and the other two orthologs match more marginally, with E< 0.75. The *nrdC.10 *ORF is conserved in 3 of 6 phages, and all 3 of these match the AAA ATPase motif, with E values ranging from 0.082 to 0.16. Another conserved ORF, *5.4*, displays a less probable, although conserved, match to the PAAR membrane associated motif. However, such low probability matches must be interpreted with caution, but they could provide starting points for the identification of the functions for conserved proteins. Functional assignments for *vs.1*, *vs.6*, and *nrdC.10 *were corroborated by BLAST matches to the Conserved Domain database [16]. In addition, Conserved Domain BLAST searches identified matches for 4 of 6 *tk.4 *orthologs to the A1pp phosphatase domain and 5 of 6 *nrdC.11 *orthologs to the COG3541 nucleotidyltransferase domain.

**Table 2:** Domain matches for T4 conserved ORFs

Gene	Pfam domain name	E value range	genomes hit
vs.6	Gly_radical formyl transferase	1.40E-45 to 8.8E-15	6/6
vs.1	SLT Transglycosylase	0.012 to 0.74	6/6
nrdC.10	AAA ATPase family	0.082 to 0.16	3/3
nrdC.10	BSD domain	0.076	1/3
nrdC.2	TFIIS_C	0.021	1/6
*nrdC.11	COG3541: nucleotidyl transferase	4.0E-07 to 0.013	2/6 full alignment 4/6 partial alignment
*tk.4	smart00506:A1pp phosphatase	2.0E-20 to 0.04	4/6 full alignment 1/6 partial alignment

Matches are HMMer matches to the Pfam database. * indicates BLAST matches to CCD database. Genomes hit shows (number of orthologs matching Pfam domain)/(total number of orthologs identified for the five genomes studied plus T4). For CDD matches, alignment to the full domain or partial length alignment is noted. Additional conserved ORFs for which no function was identified are: *uvsW.1*, *pseT.2*, *pseT.3*, *a-gt.4*, and *61.1*.

Only recently has the conserved ORF *uvsW.1 *been recognized [17] in T4. Previously this sequence was believed to encode the C-terminal 76 amino acids of the UvsW protein. For all 5 of the genomes analyzed here, the coding region corresponding to T4 *uvsW *was divided into 2 ORFs, *uvsW *and *uvsW.1*. Concurrent crystallography on the UvsW protein from T4, showed that it too lacked the region similar to *uvsW.1 *and subsequent resequencing of this region in T4 confirmed the presence of the two distinct ORFs, *uvsW.1 *and *uvsW *[17]. Although *uvsW.1 *is conserved among T4 and all 5 genomes studied here, its function remains unknown.

### Novel ORFS

Each phage genome includes a surprisingly large number of ORFs that have no matches in T4. We term these ORFs "novel ORFs" and their numbers range from 230 in Aeh1 (54% of the genome) to 62 (20% of the genome) in RB69. Similarly, 64 T4 ORFs (15% of the genome) have no apparent ortholog in RB69, its closest relative in this analysis; these 64 ORFs are novel to T4 (see Table 1). Locations of the novel ORFs appear to be non-random, with most clustered in groups between blocks of conserved genes. In a few instances, however novel ORFs are found singly between conserved genes (see Figure 1). The direction of transcription of the novel ORFs is almost invariably the same as flanking conserved genes. This suggests that the novel ORFs are subject to the same regulatory constraints as the rest of the phage genome, with early expressed genes being transcribed primarily counterclockwise and late genes being transcribed clockwise. Nearly 90% of the novel ORFs are clustered among early and middle gene orthologs, suggesting that these genes are expressed at the beginning of the infectious cycle, along with the flanking conserved genes (see also below). The novel ORFs do not appear to differ significantly in codon bias from conserved genes. They share the same strand bias of the third codon position seen in T4 [18] and do not vary significantly in codon adaptation index [19] from conserved genes (data not shown). These observations argue that the novel ORFs are not recent acquisitions of host genes.

We searched the sequences of novel ORFs for matches to phage genomes and the Swissprot database by using blastp, and Pfam motifs (HMMer). We identified a total of 750 ORFs from the 5 genomes that lacked T4 orthologs. Of these, only 64 showed matches to Pfam functional domains (Table 3) or to proteins of known function in GenBank. Although novel ORFs are not orthologs of T4-like genes, some appear to be paralogous duplications of adjacent, conserved genes, such as *RB69ORF010c *with *motB*, and *RB49ORF183c*, *44RRORF188c *and T4 ORFs *alt.-1 alt.-2*, with *alt*. An additional ORF, *44RRORF187c*, appears to be a full-length duplication of *alt*, but displays only 54% similarity to 44RR *alt*. Although none of the remaining novel ORFs showed any similarity to T4, 89 of them matched other novel ORFs from one of the other five T4-like genomes in this study. A subset of ORFs in phages 44RR, Aeh1, and RB43 appear to be orthologs of a pyrimidine salvage pathway, previously described in the T4-like phage KVP40 [7]. This pathway includes an NAPRTase and a bifunctional NUDIX hydrolase/nucleotidyl transferase, which is distinct from the monofunctional NUDIX hydrolase, *nudE*, found in T4 [20]; *nudE *orthologs were also predicted for Aeh1, RB43 and RB69. It thus appears that Aeh1 and RB43 possess both the bifunctional NUDIX protein and the T4-like monofunctional NudE protein. It is unclear whether these observations reflect a functional redundancy for RB43 and Aeh1, or if *nudE *and the bifunctional NUDIX/transferase provide different functions in the phage-infected cell. Conversely, RB49 does not appear to encode either *nudE *or the bifunctional NUDIX protein.

**Table 3:** Pfam hits for novel ORFs

**ORF**	**Pfam Domain name**	**E value**
44RRORF008c	Serine hydroxymethyltransferase	9.80E-180
44RRORF084c	TM2 domain	3.80E-14
44RRORF093c	Glutathionylspermidine synthase	8.30E-109
44RRORF097c	Prokaryotic N-terminal methylation motif	3.70E-09
44RRORF098c	SPFH domain/Band 7 family	1.10E-06
44RRORF109c	Glutaredoxin-like domain (DUF836)	0.016
44RRORF111c	Ribonucleotide reductase, small chain	4.00E-06
44RRORF130c	Prokaryotic dksA/traR C4-type zinc finger	4.30E-05
44RRORF168c	HD domain	0.34
44RRORF232c	Domain of unknown function (DUF1732)	0.35
44RRORF234c	Sodium:solute symporter family	2.60E-34
44RRORF238c	Putative metallopeptidase (SprT family)	0.33
Aeh1ORF004c	CYTH domain	0.14
Aeh1ORF010c	dUTPase	5.10E-25
Aeh1ORF025c	Carbohydrate binding domain	0.4
Aeh1ORF026c	Carbohydrate binding domain	0.12
Aeh1ORF040c	Prokaryotic N-terminal methylation motif	6.60E-09
Aeh1ORF062c	Putative metallopeptidase (SprT family)	0.00035
Aeh1ORF064c	SPFH domain/Band 7 family	2.40E-05
Aeh1ORF068c	Bacterial transferase hexapeptide (3 repeats)	0.32
Aeh1ORF110c	HD domain	0.0078
Aeh1ORF111c	UV-endonuclease UvdE	3.60E-20
Aeh1ORF131c	Poly(ADP-ribose) polymerase catalytic domain	0.026
Aeh1ORF132c	ADP-ribosylglycohydrolase	1.10E-05
Aeh1ORF154c	von Willebrand factor type A domain	0.22
Aeh1ORF157c	CreA protein	4.40E-09
Aeh1ORF227c	RyR domain	0.0054
Aeh1ORF230c	Bacterial regulatory proteins, lacI family	0.14
Aeh1ORF245c	GatB/Yqey domain	0.17
Aeh1ORF289c	Poly A polymerase family	9.00E-31
Aeh1ORF318w	Phage T4 tail fibre	8.10E-06
RB43ORF020c	LysM domain	1.70E-07
RB43ORF057w	DnaJ domain	2.70E-05
RB43ORF119c	von Willebrand factor type A domain	0.02
RB43ORF127c	C-5 cytosine-specific DNA methylase	1.20E-117
RB43ORF139c	SPFH domain/Band 7 family	3.80E-05
RB43ORF157c	PhoH-like protein PIN domain	4.20E-15 0.0032
RB43ORF179c	DnaJ central domain (4 repeats)	0.28
RB43ORF191c	DnaJ central domain (4 repeats)	0.22
RB43ORF205w	Protein of unknown function (DUF1054)	0.43
RB43ORF241c	Zeta toxin	0.36
RB43ORF282w	Phage tail fibre adhesin Gp38	0.0035
RB49ORF044c	DEAD/DEAH box helicase	0.069
RB49ORF046c	Prokaryotic N-terminal methylation motif	0.43
RB49ORF102c	D-alanyl-D-alanine carboxypeptidase	0.0014
RB49ORF143w	Methyltransferase small domain Ribosomal RNA adenine dimethylase	0.0011 0.33
RB49ORF188c	TFIIB zinc-binding	0.22
RB49ORF239c	Protein of unknown function (DUF723)	0.098
RB49ORF244c	CYTH domain	0.0026
RB49ORF260c	Protein of unknown function (DUF1311)	0.2
RB69ORF048c	Thymidylate synthase	0.022
RB69ORF050c	Peptidase family U32	0.00055
RB69ORF053c	Nucleotidyl transferase	0.0022
RB69ORF055c	SIS domain	0.0043
RB69ORF104c	Oleosin	0.42
**Putative mobile DNA elements**		
RB43ORF027c	AP2 domain	0.00071
RB43ORF066w	LAGLIDADG endonuclease	0.15
RB49ORF040c	AP2 domain HNH endonuclease	2.20E-07 0.0042
RB49ORF212c	HNH endonuclease	9.20E-07
**Putative nucleotide salvage enzymes**		
44RRORF072c	Nicotinate phosphoribosyltransferase	9.80E-63
44RRORF083c	NUDIX domain	7.10E-15
Aeh1ORF119c	Nicotinate phosphoribosyltransferase	1.30E-46
Aeh1ORF282c	NUDIX domain Cytidylyltransferase	8.30E-12 5.80E-05
Aeh1ORF330c	NUDIX domain	3.00E-08
RB43ORF138c	NUDIX domain Cytidylyltransferase	1.90E-13 5.30E-05
RB43ORF255w	Nicotinate phosphoribosyltransferase	4.50E-44

Predicted ORF protein sequences were used to search Pfam using HMMer. Matches with E < 0.5 are shown. Multiple matches are shown for ORFs having non-overlapping matches to more than one domain.

Several other novel ORFs may be involved in nucleotide modification and synthesis. These include DNA methylase, nucleotidyl transferase, nucleotide triphosphatase and sugar isomerase domain functions identified by Pfam matches. In addition, phylogenetic analyses suggest that phage 44RR appears to have acquired ribonucleotide reductase and thioredoxin genes from a bacterial host, rather than through conservation of the T4-like orthologs [13]. A number of the predicted ORFs likely to be involved in gene regulation were also identified, including DNA binding proteins, polyADP-ribosylases and -hydrolases, DNA helicases, an excision repair endonuclease and homing endonucleases, as indicated in Table 3. Other putative functions identified include membrane proteins, peptidases, ATPases, an exotoxin, and a putative DnaJ-type protein chaperone. Several ORFs that do not match known genes in GenBank do match GenBank environmental sample sequences. It is unclear if these matches are to uncharacterized bacterial hosts, or to unknown bacteriophages.

All ORFs were also searched for matches to signal peptide [21] and transmembrane motifs [22]. Tables of ORFs matching these motifs for each genome are available (see additional file 2).

### Mobile DNA elements

The T4 genome encodes a number of mobile DNA elements, including 3 group I introns with integrated ORFs encoding homing endonucleases as well as the freestanding homing endonucleases genes (HEGs), *mob *and *seg *[3]. No group I introns were detected among any of the T4-like genomes sequenced here. However, two ORFs bearing similarity to the *mob *genes of T4 were identified in Aeh1 and RB43. An ORF similar to T4 *segD *has also been described for KVP40 [7]. Thus, T4 seems to carry many more mobile elements than the genomes analyzed here. Interestingly, both RB49 and RB43 exhibit matches to a recently identified class of HEGs, AP2-HNH mobile DNA elements, which are related to the AP2 DNA transcription factor in plants [23] (also see [13]). This class of HEGs has been postulated to have transferred from bacteriophages into plant genomes via the chloroplast genome [23].

### Putative signals for transcriptional regulation

The similarities of genome organization to T4 suggested that T4 transcriptional regulatory circuits might be conserved for many T4-like phages in nature. However, phages 44RR and Aeh1 replicate in different hosts than T4 and coliphage RB43 has a substantially rearranged genome compared to the T4 prototype. The relevance of these differences to gene regulation was analyzed by prediction of transcriptional promoter elements in each genome. Consensus nucleotide sequences have been described for three temporal classes of promoters in T4: genes expressed early, middle and late in the infectious cycle [3]. Each of the five T4-like genomes was searched for matches to these T4 transcriptional regulatory signals.

### Early promoters

**Figure 2:**
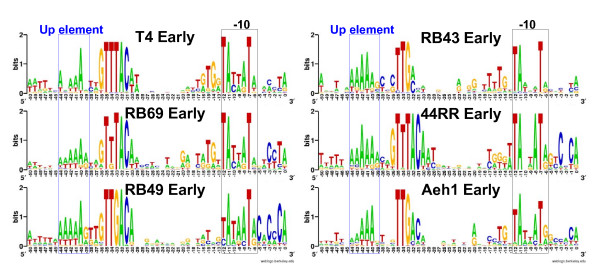
**Sequence logo representation of putative early promoter consensus for each genome**. Sequences were identified using fuzznuc [24] and HMMer [53]. Consensus sequences were plotted with WebLogo [54]. Height of letter indicates degree of conservation. Nucleotide 0 is the putative transcription start site. Putative up elements and the -10 region are boxed.

The T4 early promoter consensus [3] was used as a start point for identifying sequence similarities in the 5 T4-like genomes using the string search program fuzznuc [24]. Matching sequences were scrutinized for their locations relative to the predicted translation initiation site of putative early genes or other ORFs. These sequences were then used in an iterative fashion to find additional sequences using the HMMer program, which develops a statistical model for the consensus with which more refined searches of the genome can be done. Successive rounds of sequence selection and refinement were done until the number and locations of the sequences found ceased to change. From this analysis, we derived an early gene promoter motif for each phage. The locations of the final set of putative promoters on the genome were then manually examined. In virtually all cases, putative promoter elements were identified 5' to a predicted translational start site for a predicted ORF or conserved gene and in the correct orientation for transcription of this ORF. Thus, the predicted promoters appear to be plausible transcription initiation sequences. In each case, the sequences of the presumed early promoters thus identified had similarities to the T4 early consensus, but with some distinct differences that are illustrated in Figure 2. All predicted early promoters had similarity in the -35 region sequence to the GTTTAC sequence (-36 to -31) found in T4 [3], but in RB49, RB43 and Aeh1 there was a definite preference for G rather than T at position -33. In T4, this position is believed to be a preferred site of interaction of the ADP-ribosylated alpha subunit of RNA polymerase; a modification that is made in this subunit by the T4 encoded Alt protein [25]. Phages RB49 and Aeh1 have putative *alt *genes, but in both cases the predicted Alt protein sequences are considerably diverged from the T4 sequence (data not shown); RB43 apparently lacks an *alt *ortholog. Position -36 is a strongly conserved G in some of the genomes analyzed but for RB43 it can be G or C; Aeh1 shows even less sequence conservation in the -36 position. All the phages frequently have an A-rich sequence from -40 to -44. This region resembles the UP element, which enhances transcription and is a site of interaction with the T4 ADP-ribosylated alpha subunit of RNA polymerase [26-28].

All putative early promoters resemble the T4 consensus in the -10 region, which is recognized in the host by the σ subunit of RNA polymerase. In general, there is high conservation of T at position -7 and A residues at position -11, as seen in T4. However, in our phage conservation of the T at position -12 is variable; T is not rigidly conserved at position -12 in Aeh1, and in RB49 it can be either T or C. There is variable conservation of the GT-rich sequence 5' to position -12 exhibited by T4. 44RR shows a higher degree of conservation of A at -8 than any of the other phages. The genomes of RB69, RB49, and 44RR all show preference for C residues in the -3 to -1 region. The predicted RB49 early consensus agrees with that previously identified by 5' end mapping of RB49 early transcripts [29].

**Figure 3:**
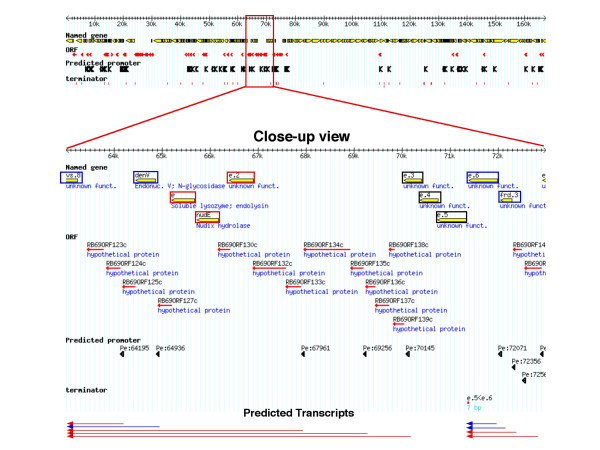
**Location of early promoter sequences on the RB69 genome**. The top panel shows an overview. Conserved Genes are shown as yellow arrows, novel ORFs as red line arrows, predicted early promoters are shown as large black arrows, and TransTerm [38] predicted terminators as red blocks. The bottom panel shows detail of one region. Predicted transcripts are shown at the bottom, blue arrows indicate transcripts expected from conserved gene promoters and red arrows designate those expected from novel ORF promoters. Orthologs of genes known to be expressed early in T4 infections are boxed. Red boxes indicate genes present only on predicted ORF promoter transcripts; blue-boxed genes are present on conserved and ORF promoter transcripts. Black boxes are early genes whose transcripts could not be predicted.

When the sites of predicted early promoters were mapped onto their respective genomes, many promoters were located 5' to orthologs of T4 early genes, as expected. Importantly, a large number of early promoters were predicted 5' to novel ORFs, including those for which no homologs exist in the sequence databases. For example, of 57 putative early promoters in RB69, 13 were upstream of novel ORFs and 45 were upstream of T4 orthologs (see example in Figure 3). These observations suggest that many novel ORFs are coordinately regulated along with the flanking conserved early T4-like genes. Early promoters were also found 5' to the tRNA genes, described below. Coordinates of putative early promoters can be found in the supplements (see additional file 3).

### Middle promoters

**Figure 4:**
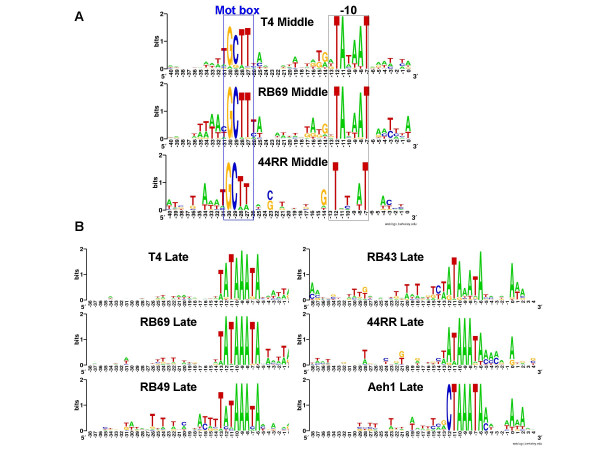
**(A) Sequence logo representation of putative middle promoter consensus for RB69 and 44RR**. Consensus was identified and plotted as in Figure 2. **(B) **Putative late promoter consensus for each genome. Consensus was identified as for early promoters, using fuzznuc and HMMer, except Aeh1, for which ELPH [37] and HMMer were used initially.

In the T4 infectious cycle, early transcription is followed by "middle mode" transcription, which is initiated by the binding of the phage-encoded MotA protein to its cognate recognition sequence at T4 middle promoters [30-32]. We used two criteria to attempt to detect conserved elements of T4-like middle mode transcriptional regulation among the five genomes studied: (a) matches to the T4 middle promoter consensus [33] and (b), matches to the T4 MotA protein sequence. The RB69 genome includes a *motA *ortholog (blastp E = 5X10^-48^). Putative RB69 middle promoter sequences were identified using a similar strategy to that described for early promoters, but based upon the consensus sequence, (a/t)(a/t)(a/t) TGCTTtAN(11–13)TataAT [33] The RB69 middle consensus clearly resembles that of T4 (Figure 4A); with conservation of the residues at positions -12, -11, and -7 of the T4 consensus. Also, the putative RB69 middle genes exhibit extended conserved sequences from positions -13 to -16, as seen in T4. T4 middle promoters show little similarity to the -35 region of *E. coli *σ^70 ^promoters, but do possess the highly conserved GCTT motif (the T4 Mot box) at positions -30 to -27. This motif serves as the site of interaction of the T4 MotA protein with DNA. RB69 middle promoters also show similarity to the Mot box, which is presumably bound by the RB69 MotA ortholog. However, among the 4 other genomes studied, only the 44RR genome had an ortholog to the T4 MotA protein and sequence motifs similar to the T4 MotA-dependent promoters. Nine putative 44RR middle promoters were identified. They resemble the middle-mode consensus sequences of both T4 and RB69, but lack conservation at nucleotide position -11 (Figure 4A). The relatively small number of putative middle-promoters that we have detected in 44RR tempers the interpretation of their significance. However, the presence of a strong match (blastp E = 2X10^-33^) to the T4 *motA *gene function in this *Aeromonas *phage is probably indicative of the presence of a 44RR-encoded middle-mode transcriptional apparatus. Previous attempts to identify a middle promoter consensus and a *motA *ortholog in RB49 were unsuccessful [29] as were our attempts for RB49, RB43 and Aeh1. RB69 and 44RR also possess orthologs of the MotA co-activator AsiA [34]. Surprisingly, Aeh1 and KVP40, also encode AsiA proteins, which have been shown to bind T4 MotA [35], even though no ligand homologous or analogous to MotA has been identified for these genomes. AsiA can act as transcriptional inhibitor in the absence of MotA [35], or may interact with another phage protein which has yet to be identified. Coordinates of putative middle promoters can be found in the supplements (see additional file 4).

### Late promoters

In T4, late promoters are recognized by a phage-encoded σ factor, gp55. Contact between T4 gp55 and the DNA is facilitated by the T4 polymerase sliding clamp, gp45. A third T4-encoded gene product, gp33 forms a bridge between gp55 and gp45 [36]. The T4 late promoter consensus sequence is a short but highly conserved motif, TATAAATA, between nucleotide positions -13 and -6 relative to the transcriptional start site [3]. Putative late promoters were found readily for four of the five phage genomes studied, using the strategy employed for early and middle promoter searches (Figure 4B). However, the T at position -13 was poorly conserved for most phages, with either A or T commonly found at this position. A similar observation was made for late promoters in an earlier description of RB49 late promoters [29], as well as in KVP40 [7] and S-PM2 [8].

Since our search strategy failed to detect late promoter sequences for phage Aeh1, an alternative strategy was employed to identify them. Regions upstream of ORFs orthologous to T4 late genes were analyzed with the ELPH program [37] to identify sequence motifs common to these DNA segments. The selected motifs were used as seed to identify additional late promoter sequences using HMMer. This strategy identified a conserved sequence, CTAAATA, beginning at -12 from the putative initiation site. Once identified, this putative promoter sequence was used as a seed for string search followed by HMM refinement used for late promoters of the other phages. Although the C at position -12 is a strong determinant for detection of Aeh1 late promoters, C is rarely found at this position in the putative late promoters of the other four phage genomes (Figure 4B). It should be noted that the phage Aeh1 gp55 protein, which presumably recognizes the divergent late promoter sequences of Aeh1, is itself substantially diverged from all the other phage gp55 sequences (data not shown). Coordinates of putative late promoters can be found in the supplements (see additional file 5).

### Terminators and operons

Putative rho-independent terminator sequences were identified for all 5 genomes, using the TransTerm program [38]. Although the locations of putative terminator sequences vary between phages, several terminators appear at conserved locations (see additional file 6). One striking example is the bi-directional terminator predicted downstream of *uvsW.1*that is conserved in T4 and the other 5 genomes. In all cases, the gene downstream of *uvsW.1 *is transcribed from the opposite strand and a bidirectional terminator is predicted between the converging transcripts. Genes *35 *and *36 *are transcribed rightward and a predicted terminator is located between them in all 6 genomes. Likewise, gene *23 *has a terminator predicted downstream in all 6 genomes. Terminators conserved in 5 out of 6 genomes were identified downstream of Gene *32 *and upstream of *alt*.

Comparisons between the positions of predicted terminators and transcription initiation signals allowed the identification of putative operons of gene expression. An example of operon structure from phage RB69 is shown in Figure 3. In some instances, it appears that the upstream promoters of novel genes drive expression of T4-like early genes that lack their own early promoter. In general, T4-like genes are predicted to be in operons with other T4-like genes, while novel ORFs appear to reside in operons with other novel ORFs.

### tRNAs and codon bias

**Figure 5:**
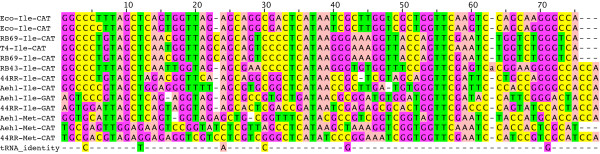
**tRNA alignment**. Putative lysidine-modified phage tRNA-Ile sequences were aligned by secondary structure using clustalW. E. coli modified tRNA-Ile and phage Met-CAU and Ile-GAU sequences are shown for comparison.

The bacteriophage T4 genome encodes eight tRNA genes [3]. The other T4-like genome sequences were searched for potential tRNA genes, using tRNAscan-SE [39]. The number of potential tRNA genes varied considerably among genomes (Table 1), ranging from zero in RB49 to 24 in Aeh1. Some common features were noted among the tRNA genes encoded by the phage genomes (Table 4). All genomes that encoded tRNAs had a predicted tRNA with a CAU anticodon. Although predicted to be Met tRNA by tRNAscan-SE, these tRNAs share signature sequences found in tRNAs recognized by IleRS [40]. This class of Ile tRNAs is post-transcriptionally modified to lysidine at the anticodon, converting them to Ile-recognizing anticodons resembling AUA [41-44]. An alignment of phage Ile and Met tRNAs is shown in Figure 5. tRNAs for Leu, Ser and Arg are among the most commonly identified putative tRNAs genes encoded in the T4-like genomes, including the previously sequenced genomes of T4 and KVP40. Other tRNAs are found more rarely, such as Ala, Pro, Gly and Val. These recognize GC rich codons, which are unusual in AT-rich T4-like genomes [18].

**Table 4:** Predicted tRNAs

**tRNA**	**Aeh1**	**44RR**	**T4**	**RB69**	**RB43**
Ala UGC	+	+			
Arg UCU		+	+		
Asn GUU	+	+			
Asp GUC	+	+			
Cys GCA	+				
Gln UUG			+		
Glu UUC	+				
Gly UCC	+		+	+	
His GUG	+	+			
Ile CAU*	+	+	+	+	+
Ile GAU	+	+			
Leu CAA	+				
Leu UAA	+	+	+		
Leu UAG	+				
Lys UUU	+	+			
Met CAU	+	+			
Met CAU	+				
Phe GAA	+	+			
Pro UGG	+	+	+		
Ser GCU		+			
Ser UGA	+	+	+		
Thr UGU	+	+	+		
Trp CCA	+	+			
Tyr GUA		+			
Val CAC	+				
Pseudo	3	1			

The presence of a tRNAscan-SE predicted species is indicated for each genome. The number of predicted tRNA pseudogenes is also indicated. * indicates putative lysine-modified tRNA^Ile ^[41-44].

In bacteriophage T4, the presence of tRNA genes appears to correlate with differences in codon bias for the phage versus the *E. coli *host [3]. The genomes sequenced here show much less correlation to differences from their laboratory hosts. A similar observation was made for the vibriophage KVP40 [7]. Thus, the functional role of the tRNA genes for these phages remains unclear. Nevertheless, the high degree of conservation of some tRNAs, such as the putative modified tRNA^Ile ^mentioned above, suggests an important functional role for at least some of these tRNAs.

## Discussion

The genome sequences presented here display broad diversity in primary sequence. Orthologous ORFs can be detected for 45 to 85 percent of open reading frames between any pair of these genomes. Orthologous protein sequences are on average 65% similar between genomes. This diversity is comparable to that seen across vertebrate evolution. For example, humans and chickens share 60% orthologous genes at a median amino acid similarity of 75%. Humans and teleost fishes share approximately 55% orthologous genes. The two most closely related phage genomes analyzed here, T4 and RB69, share 80% orthologs of 81% similarity, a distance comparable to that between humans and mice. Despite the diversity of their predicted protein sequences, these five T4-like phage genomes share a highly conserved genome organization. Most orthologs of T4 genes were identified in the same gene order and orientation as the cistrons in T4. RB43 shows the largest number of exceptions to this observation. It appears that several genome rearrangements must have occurred in one or both of these phages since they diverged from their common ancestor.

The possibility of shared genetic regulatory elements among the T4-like phages was investigated by motif searches that identified putative promoter elements resembling T4 early and late promoters in all genomes. Late promoters were found exclusively 5' to conserved orthologs of T4 late genes. Many early promoters were found 5' to T4 early gene orthologs, but others were found 5' to novel ORFs. It thus appears that the early and late transcriptional modes are conserved among the T4-like phages. The novel ORFs appear to be coordinately expressed with early genes in all phages. The middle gene expression pathway appears to be less conserved among the T4-like phages. The middle promoter consensus was detected in RB69, and to a lesser degree in 44RR. The MotA protein product, required for recognition of the middle promoter Mot box, appears to be conserved only in T4, RB69 and 44RR.

The T4 genome is predicted to encode over 120 ORFs of unknown function. 11 ORFs were found to have homologs in all five of the genomes in our study. Given this level of conservation, these ORFs must encode products that are vital to the phage in some hosts or environments. We have identified putative functional domains for 5 of these ORFs based on matches to known Pfam domains. The candidate functions include nucleotide metabolism, host cell lysis, and gene regulation. An aggregate of about 70% of T4 ORFs are conserved in at least one other genome, suggesting that the protein products of these ORFs provide selective advantages to these phages. Conservation of these ORFs does not generally extend to more divergent phages than those analyzed here. Although several of these ORFs are conserved in KVP40, no matches were found in any of the marine phage genomes.

Each of the T4-like genomes we have examined, including T4, harbors a number of ORFs that are unique to that genome. In Aeh1, these novel ORFs comprise over half of the Aeh1 genome and most show no significant similarity to known sequences in GenBank. Functions identified for some novel ORFs suggest physiologically important roles in the phage life cycle, such as nucleotide metabolism, transcription and lateral DNA mobility. However, most novel ORFs have no known function or origin. It is thus unclear where these sequences arose, how they were acquired, and what function they might serve in the phage-infected cell. In many instances, regions containing novel ORFs were observed to be underrepresented in plasmid libraries constructed for shotgun sequencing and were only identified during PCR-based gap closure [7] and data not shown). It would appear then, that at least some novel ORFs in our study are deleterious to the host cell when expressed in high copy plasmids. Some of the gene products of these ORFs may function in cell lysis or in commandeering host machinery for phage growth.

The mechanisms of gain and loss of ORFs by T4-like genomes in evolution may differ from that proposed for the genomes of other phages, such as the lambdoid phage [45]. The novel lambdoid ORFs include "morons" – apparent short insertions of DNA consisting of an ORF flanked by transcriptional promoter and terminator signals. Moron DNAs are distinct from other lambdoid genes in %GC content, and thus appear to be recent acquisitions of genes by nonhomologous recombination with host DNA. In contrast, the majority of novel ORFs in T4-like phages does not appear moronic; they have a %GC that is indistinguishable from the rest of the phage genome (average %GC in RB69: ORFs-36.9%, conserved-37.6%) and thus do not appear to be recent acquisitions from the host. Another class of novel lambdoid ORFs appears to be chimeras of other phage genes [46]. In the few instances where the T4-like novel ORFs have significant matches to other phage or GenBank proteins, the similarities generally extend over the entire length of the coding sequence rather than being restricted to the blocks of similarity found in chimeras. A better understanding of the origins of the novel ORFs in T4-like phages will provide clues into the mechanisms underlying the evolution of protein coding sequences and the biology of host-phage interactions. The mechanisms by which T4-like phages acquire ORFs may differ from the lambdoid phages. T4-like phage do not undergo lysogeny, thus they cannot acquire genes by imprecise excision from the host genome. They do not generally transduce host DNA as frequently as other Myoviridae, such as P22 [47], perhaps because of their propensity to hydrolyze host DNA. T4-like phages have a recombination-driven replication pathway that is facilitated by redundant DNA sequences at the chromosome ends. During replication, the redundant end sequences synapse with homologous regions of other replicating DNA molecules for further replication into long concatamers [6]. A variation of this pathway has been postulated as a mechanism for the lateral transfer of novel genes between related phages [48]. However, the ultimate source of these novel genes remains unknown but may include bacterial hosts or bacteriophages encountered in coinfection. The failure to detect significant similarities between many of the novel ORFs described here and known bacterial genomes indicates that either these ORFs arose from bacterial hosts quite diverged from any known bacterium, or that bacterial genomes are not a major source for these ORFs. The latter appears to be more likely, at least in the case of novel ORFs identified in closely related phages, such as T4 and RB69. Unknown phages would seem a more likely source for many of these ORFs. Newly sequenced phage genomes often include numerous ORFs for which there is no known ortholog. Clearly, more phage genomes must be mined to incorporate more of their sequence diversity into the library of known sequence databases.

## Conclusion

Our survey of a diverse set of T4-like phage genomes reveals similarities in general genome organization and gene regulation. Although a core of conserved ORFs was identified, the genome sequences exhibited a striking diversity of ORFs novel to each genome. The origins of this diversity have yet to be uncovered.

## Methods

### Bacteriophages and hosts

Bacteriophages, bacterial hosts and growth conditions were as described [13]. Phage DNA was prepared from plate lysates sequenced, and assembled as described in [13].

### Genome annotation

ORFs were detected primarily by use of the GeneMarkS program [11,12]. The program was chosen based upon its accuracy in ORF prediction of the T4 genomic sequence by comparison to the GenBank accession (97% of ORFs recognized). When an orthologous gene was detected in a related phage genome, the predicted translational start sites were scrutinized for additional N-terminal protein sequences with significant similarity to orthologs upstream of the predicted translational start site. In these cases, the translational start site was adjusted to maximize the length of predicted amino acid similarity. Although prediction models were not based upon similarity between genomes, generally fewer than 5% of the predicted start sites required adjustment.

GeneMarkS predictions were compared with those obtained using Glimmer [49]. There was general agreement between the predictions obtained with the two programs. Glimmer predicted more ORFs per genome, but in some cases the additional ORFs predicted were inconsistent with the direction of transcription of flanking genes, which is uncommon in T4 [3] and appears unusual for the genomes sequenced here. Thus, the Glimmer predictions were used primarily to adjust GeneMarkS predictions as mentioned above, or in regions where Glimmer predicted an ORF and GeneMarkS predicted an unusually long (> 200 bp) intercistronic region.

Predicted ORFs were checked for similarity to T4 genes by blastp [50] mutual similarity. Genes with mutual best hit E-values < 10^-4 ^to known T4 genes were designated by the T4 gene name. Putative genes without T4 orthologs were designated by their ORF numbers, with conserved gene *rIIA *designated as ORF001. The strand of each ORF is designated "w" for clockwise (left-to-right) transcribed genes, and "c" for counterclockwise (right-to-left) transcribed genes. In T4, the origin of the genome has been assigned to the *rIIB *– *rIIA *intercistronic region; the terminus of the genome is defined as the start of translation of the *rIIB *gene. The sequence origin of each genome sequenced here is defined as the termination codon of the *rIIA *gene.

Genomes were also searched for tRNA genes using tRNAscan-SE [39]. All genomes except that of RB49 had at least one putative tRNA gene.

DNA sequences are available through GenBank [Genbank:NC_005135] (44RR), [Genbank:NC_007023] (RB43), [Genbank:NC_004928] (RB69), [Genbank:NC_005260] (Aeh1), and [Genbank:NC_005066] (RB49). Additional analyses are available through the Tulane T4-like Genome Website  Available data include an interactive genome browser [51], clustalW [52] alignments, EMBOSS pepstat statistics, octanol hydropathy plots [24], and HMMer Pfam matches [53].

## Competing interests

The author(s) declare that they have no competing interests.

## Authors' contributions

JMN designed and performed machine annotations of all genomes, performed promoter searches and drafted the manuscript and figures. VP provided additional annotations for all genomes and aided in construction of annotation tables. CB contributed to annotations. HMK co-conceived the study and provided manuscript comments. JDK conceived of the study, and participated in its design and coordination and helped to draft the manuscript. All authors read and approved the final manuscript.

## Supplementary Material

Additional File 1 **High-resolution genome map**. Genome map is as indicated for Figure 1, but predicted gene names are also indicated. Click here for file

Additional File 2 **Predicted transmembrane and signal peptide matches for ORFs**. The amino acid coordinates of each ORF matching Transmembrane [22] orSignal peptide [21] motifs are indicated for each ORF. Multiple transmembrane regions are predicted for some ORFs. Click here for file

Additional File 3 **Coordinates of predicted early promoters**. Coordinates are in GFF format. For promoters on + strand, the 5' end of the sequence is the leftmost coordinate, for promoters on – strand, the 5' end of sequence is the rightmost coordinate. Promoters are named by their 5' end; those that differ in length from the consensus are noted. Click here for file

Additional File 4 **Coordinates of predicted middle promoters**. Coordinates are in GFF format. For promoters on + strand, the 5' end of the sequence is the leftmost coordinate, for promoters on – strand, the 5' end of sequence is the rightmost coordinate. Promoters are named by their 5' end; those that differ in length from the consensus are noted. Click here for file

Additional File 5 **Coordinates of predicted late promoters**. Coordinates are in GFF format. For promoters on + strand, the 5' end of the sequence is the leftmost coordinate, for promoters on – strand, the 5' end of sequence is the rightmost coordinate. Promoters are named by their 5' end. Click here for file

Additional File 6 **Coordinates of Predicted rho-independent terminators**. Coordinates are in GFF format. For promoters on + strand, the 5' end of the sequence is the leftmost coordinate, for promoters on – strand, the 5' end of sequence is the rightmost coordinate. Bidirectional terminators have the strand designation "." Terminators are named according to their flanking genes. Click here for file
