# Supplementary material for: Genetic diversity among five T4-like bacteriophages
Source: Virol J. 2006 May 23;3:30. doi: 10.1186/1743-422X-3-30 (PMC1524935; doi:10.1186/1743-422X-3-30)
Supplement: Additional File 1 — High-resolution genome map. Genome map is as indicated for Figure 1, but predicted gene names are also indicated. [file 1743-422X-3-30-S1.pdf]

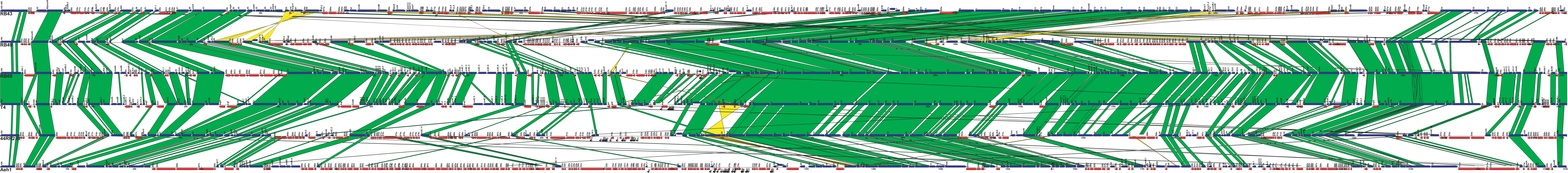
RB43
RB49
RB69
T4
44RR2.8t
Aeh1
